# Mutation analysis of the c-mos proto-oncogene and the endothelin-B receptor gene in medullary thyroid carcinoma and phaeochromocytoma.

**DOI:** 10.1038/bjc.1996.363

**Published:** 1996-08

**Authors:** C. Eng, K. A. Foster, C. S. Healey, C. Houghton, S. A. Gayther, L. M. Mulligan, B. A. Ponder

**Affiliations:** CRC Human Cancer Genetics Research Group, University of Cambridge, Addenbrooke's Hospital, UK.

## Abstract

The characteristic tumours of MEN 2 are medullary thyroid carcinoma (MTC) and phaeochromocytoma. Somatic RET mutations have been found in only 23-40% of sporadic MTC and 10% of sporadic phaeochromocytomas. Thus, we sought other genes which may play a role in the pathogenesis of these tumours. We carried out direct sequence analysis of human c-mos and human ENRB in a series of sporadic MTC and phaeochromocytomas to determine if somatic mutations in these two genes could account for some of the sporadic MEN 2-related tumours in which no RET mutations are detected. No somatic mutations were found.


					
Bridsh Journal of Cancer (1996) 74, 339-341

?  1996 Stockton Press All rights reserved 0007-0920/96 $12.00             0

SHORT COMMUNICATION

Mutation analysis of the c-mos proto-oncogene and the endothelin-B

receptor gene in medullary thyroid carcinoma and phaeochromocytoma

C Eng' 2, KA Foster', CS Healey', C Houghton', SA Gaytherl, LM Mulligan3 and BAJ Ponder'

'CRC Human Cancer Genetics Research Group, University of Cambridge, Level 3, Laboratories Block, Addenbrooke's Hospital Box
238, Hills Road, Cambridge CB2 2QQ, UK; 2Division of Cancer Epidemiology and Control, Dana-Farber Cancer Institute,

Department of Medicine, Harvard Medical School, M3A.31, 44 Binney Street, Boston, MA 02115-6084, USA; 3Departments Of

Pathology and Paediatrics, Queen's University, 20 Barrie Street, Kingston, ON K7L 3N6, Canada.

Summary The characteristic tumours of MEN 2 are medullary thyroid carcinoma (MTC) and
phaeochromocytoma. Somatic RET mutations have been found in only 23-40% of sporadic MTC and
10% of sporadic phaeochromocytomas. Thus, we sought other genes which may play a role in the pathogenesis
of these tumours. We carried out direct sequence analysis of human c-mos and human ENRB in a series of
sporadic MTC and phaeochromocytomas to determine if somatic mutations in these two genes could account
for some of the sporadic MEN 2-related tumours in which no RET mutations are detected. No somatic
mutations were found.

Keywords: MEN 2; RET; c-mos; endothelin-B receptor; medullary thyroid carcinoma; phaeochromocytoma

Multiple endocrine neoplasia type 2 (MEN 2) is an
autosomal dominantly inherited cancer syndrome associated
with germline mutations in the RET proto-oncogene, which
codes for a receptor tyrosine kinase expressed in tissues and
tumours of neural crest origin (Bolino et al., 1995; Carlson et
al., 1994; Donis-Keller et al., 1993; Eng et al., 1994, 1995c;
Hofstra et al., 1994; Mulligan et al., 1993, 1994, 1995). MEN
2 is characterised by the presence of MTC and phaeochro-
mocytoma. Somatic RET mutations have been detected in
23-40% of sporadic MTC in series comprising ten or more
tumours (Eng et al., 1994, 1995b; Hofstra et al., 1994;
Komminoth et al., 1995; Zedenius et al., 1994) and 10-20%
of sporadic phaeochromocytomas (Beldjord et al., 1995; Eng
et al., 1994, 1995a; Lindor et al., 1995).

The human c-mos proto-oncogene encodes a serine-
threonine protein kinase expressed at high levels in germ
cells (reviewed in Yew et al., 1993). It is required for meiosis
and plays a role in the initiation of oogenesis (Colledge et al.,
1994; Sagata et al., 1988). Transgenic mice ectopically
overexpressing mos develop medullary thyroid carcinomas
(MTC) and phaeochromocytomas (Schulz et al., 1992). The
endothelin-B receptor gene (ENRB) codes for a G-protein-
coupled receptor, which is expressed in several tissues,
including the kidneys, adrenal medulla and phaeochromocy-
tomas (reviewed in Davenport, 1996; Davenport et al., 1994).
Recently, germline mutations in ENRB have been found in
patients with Hirschspung disease (HSCR) (Puffenberger et
al., 1995), a common congenital condition characterised by
the lack of enteric ganglia leading to intestinal obstruction
(Okamoto and Ueda, 1967). Up to 40% of patients with
HSCR have mutations in the RET proto-oncogene (Attie et
al., 1995).

To determine if mutations in the c-mos or ENRB genes
play a role in the pathogenesis of the sporadic tumours in
which no RET mutations have been detected, the coding
sequence of c-mos and ENRB was examined in DNA from
ten sporadic MTC and ten sporadic phaeochromocytomas
which do not have known somatic RET mutation.

Materials and methods
Tumours

The ten MTCs and nine of ten phaeochromocytomas studied
here have been described previously (Eng et al., 1994,
1995a,b). The remaining phaeochromocytoma was a uni-
lateral tumour occurring in a patient without first- or second-
degree relatives with these syndromes, known phaeochromo-
cytoma or MTC, or other stigmata of MEN 2/von Hippel-
Lindau disease. Genomic DNA was extracted as described by
Mathew et al. (1987).

Polymerase chain reaction (PCR) and DNA sequence analysis
PCR was performed, using 50- 100 ng of template genomic
DNA and red hot Thermus icelandicus DNA polymerase,
according to the manufacturer's recommendations (Advanced
Biotechnologies, Surrey, UK) in the presence of 1.5 mM
magnesium chloride.

A total of 30 to 40 cycles of amplification were carried out
at 95?C for 1 min, 59?C (c-mos) or 60?C (ENRB) for 1 min
and 72?C for 1 min, followed by a final 10 min cycle at 72?C.
For c-mos, primer pairs used were Hu Mos IF (5'-TC-
TTCATTCACTCCAGCGG-3') and Hu Mos IR (5'-AAGT-
CGCCTTGTACACCGAG-3'), Hu Mos 2F (5'-GGTGTG-
CTTGCTGCAGAG-3') and Hu Mos 2R (5'-CGCCATA-
GATGACTTGGTGT-3'), Hu Mos 3F (5'-CCTAGGGAC-
CATCATCATGG-3') and Hu Mos 3R (5'-GTGTCTGG-
AAGCACAGCAGA-3') or Hu Mos 4R (5'-GGACGGGCG-
CAGGTCGTAGGCCAC-3'), and Hu Mos 4F (5'-TCAGT-
GAGCAGGATGTCTGTAA-3') and Hu Mos 5R (5'-CT-
TGACCAAGTTTTCAGTCAGC-3'). For ENRB, the primer
pairs used were ETB-OF (5'-CACACCCCTTCCAGAACG-3')
and ETB-1R (5'-CGCCCTTTACCTTGTAGACATT-3')
[exon 1], ETB-2Fa (5'-CTGCTGGCAGAGGACTGG-3')
and ETB-13R (AAGGAGTGGGGAACAGGG-3') [exons 2
and 3], ETB-44F (5'-GCTATGAGTAAAATGAGCCAT-3')
and ETB-15R (5'-CTGCCTATAAAAAAGATCGATGG-3')
[exon 5], ETB-5F (5'-CAGGATTCTGAAGCTCACTCTTT-
3') and ETB-46R (5'-TGTTTAAAAAAATCATCCATGA-3')
[exon 6], and ETB-16F (5'-ATACAAAGAAAGTCAGAA-
CCCTGG-3') and ETB-8R (5'-TTTTTGTTTTGTTTTGG-
CAAAT-3') [exon 7]. Primers for exon 4 have been previously
described (Puffenberger et al., 1995).

PCR products were gel and column purified (Eng et al.,

Correspondence: C Eng, Division of Cancer Epidemiology and
Control, M3A.31, Dana-Farber Cancer Institute, 44 Binney Street,
Boston, MA 02115-6084, USA

Received 1 December 1995; accepted without revision 15 February
1996

c-mos and ENRB mutatdons in MTC and phaeochromocytoma
$0n                                                             C Eng et at

IAO

1994). Twenty aliquots of 100 ng of PCR product served as
the template for cycle sequencing (Cyclist kit or the Cyclist
Exo-Pfu DNA sequence kit, Strategene). Sequence variants
were confirmed by digestion with an appropriate restriction
enzyme.

Results

No somatic mutations were detected in the coding sequence
of c-mos or the endothelin-B receptor gene among ten
sporadic MTC and ten sporadic phaeochromocytomas. In
addition, all PCR products were the expected sizes,
suggesting no deletions or splicing variants which could
have been missed by sequence analysis.

A novel sequence polymorphism was detected within the
kinase domain of c-mos: a G (A1) to T (A2) conversion,
changing an alanine to a seine at codon 105. This causes loss
of a BlpI restriction site. The Al and A2 alleles occurred at
frequencies of 0.85 and 0.15, respectively, among 122
Caucasian chromosomes.

Discussion

Although the tumours that developed in transgenic c-mos
mice resembled those of MEN 2, suggesting that the human
homologue may be involved in MEN 2-related tumours, no
somatic c-mos mutations or rearrangements could be found
in human MTC or phaeochromocytomas. However, gene
amplification in the tumours could not be excluded with our
analyses, and sufficient DNA was not available for gene
dosage studies. The lack of c-mos mutations in human
tumours might be because in transgenic mice, the mos
constructs were transcribed from the Moloney murine
sarcoma virus LTR, a relatively strong promoter which

References

ATTIE T, PELET A, EDERY P, ENG C, MULLIGAN LM, AMIEL J,

BOUTRAND L, BELDJORD C, NIHOUL-FEKETE C, MUNNICH A,
PONDER BAJ AND LYONNET S. (1995). Diversity of RET proto-
oncogene mutations in familial and sporadic Hirschsprung
disease. Hum. Mol. Genet., 4, 1381 - 1386.

BELDJORD B, DESCLAUX-ARRAMOND F, RAFFIN-SANSON M,

CORVOL J-C, DE KEYSER Y, LUTON J-P, PLOUIN P-F AND
BERTAGNA X. (1995). The RET proto-oncogene in sporadic
pheochromocytomas: frequent MEN 2-like mutations and new
molecular defects. J. Clin. Endocrinol. Metab., 80, 2063-2068.

BOLINO A, SCHUFFENECKER I, LUO Y, SERI M, SILENGO M,

TOCCO T, CHABRIER G, HOUDENT C, MURAT A, SCHLUMBER-
GER M, TOURNIAIRE J, LENOIR GM AND ROMEO G. (1995).
RET mutations in exons 13 and 14 of FMTC patients. Oncogene,
10, 2415-2419.

CARLSON KM, DOU S, CHI D, SCAVARDA N, TOSHIMA K,

JACKSON CE, WELLS SA, GOODFELLOW PJ AND DONIS-
KELLER H. (1994). Single missense mutation in the tyrosine
kinase catalytic domain of the RET protooncogene is associated
with multiple endocrine neoplasia type 2B. Proc. Natl Acad. Sci.
USA, 91, 1579-1583.

COLLEDGE WH, CARLTON MBL, UDY GB AND EVANS MJ. (1994).

Disruption of c-mos causes parthenogenetic development of
unfertilized mouse eggs. Nature, 370, 65- 68.

DAVENPORT AP. (1996). Distribution of endothelin receptors. In

Endothelins in Biology and Medicine, Miller R, Pelton JT and
Huggins J. (eds) CRC Press: Florida (in press).

DAVENPORT AP, MAGUIRE JJ AND KARET FE. (1994). Endothelin

receptors and their subtypes. In Endothelin: Role in Health and
Disease, Gulati A. (ed.) pp. 17 -30. Harwood Academic Publish-
ers: Amsterdam.

DONIS-KELLER H, DOU 5, CHI D, CARLSON KM, TOSHIMA K,

LAIRMORE TC, HOWE JR, MOLEY JF, GOODFELLOW P AND
WELLS SA. (1993). Mutations in the RET proto-oncogene are
associated with MEN 2A and FMTC. Hum. Mol. Genet., 2, 851 -
856.

may also cause ectopic patterns of expression; or because of
species-specific differences. Finally, it is possible that c-mos
may play a role in rare MTC and phaeochromocytomas but
the numbers examined precluded detection. In light of our
data, it is interesting to note that in vitro, many deletion or
missense mutations of conserved residues, except for one 10-
residue deletion, in Xenopus mos result in the elimination of
biological and kinase activity (Fukasawa et al., 1995).

The finding of germline mutations of ENRB in HSCR and
its expression pattern suggested that this gene would be a
good candidate for involvement in the pathogenesis of MTC
and phaeochromocytoma. However, no mutations were
detected. Thus, mutation of neither human c-mos nor ENRB
appears to play a major role in the tumorigenesis of MTC
and phaeochromocytomas.

A novel sequence polymorphism in the kinase domain of
c-mos was detected. The polymorphisms could serve as a
useful marker in the region of human c-mos on chromosome
subband 8ql2, and may in addition help in the understanding
of computer-modelled structure - function relationships of
kinase domains.

Acknowledgements

We thank Dr Darrin P Smith for critical review of this manuscript.
CE is grateful to Dr Jan Vijg for his continued support and
encouragement. This work was supported by core and programme
grants from the Cancer Research Campaign [CRC], the CRC
Dana-Farber Cancer Institute Fellowship (CE), the Susan and
Larry Marx Fellowship in Cancer Genetics (CE), the Dana-Farber
Cancer Institute (CE), a National Science Foundation Graduate
Research Fellowship (KAF), the Overseas Research Student
Awards Scheme (KAF), an MRC-Canada Operating Grant
(LMM) and the Kingston Hospital Foundation (LMM). BAJP is
a Gibb Fellow of the CRC.

ENG C, SMITH DP, MULLIGAN LM, NAGAI MA, HEALEY CS,

PONDER MA, GARDNER E, SCHEUMANN GFW, JACKSON CE,
TUNNACLIFFE A AND PONDER BAJ. (1994). Point mutation
within the tyrosine kinase domain of the RET proto-oncogene in
multiple endocrine neoplasia type 2B and related sporadic
tumours. Hum. Mol. Genet., 3, 237-241.

ENG C, CROSSEY PA, MULLIGAN LM, HEALEY CS, HOUGHTON C,

PROWSE A, CHEW SL, DAHIA PLM, O'RIORDAN JLH, TOLEDO
SPA, SMITH DP, MAHER ER AND PONDER BAJ. (1995a).
Mutations of the RET proto-oncogene and the von Hippel -
Lindau disease tumour suppressor gene in sporadic and
syndromic phaeochromocytoma. J. Med. Genet., 32, 934-937.

ENG C, MULLIGAN LM, SMITH DP, HEALEY CS, FRILLING A,

RAUE F, NEUMANN HPH, PFRAGNER R, BEHMEL A, LORENZO
MJ, STONEHOUSE TJ, PONDER MA AND PONDER BAJ. (1995b).
Mutation of the RET proto-oncogene in sporadic medullary
thyroid carcinoma. Genes Chrom. Cancer, 12, 209 - 212.

ENG C, SMITH DP, MULLIGAN LM, HEALEY CS, ZVELEBIL MJ,

STONEHOUSE TJ, PONDER MA, JACKSON CE, WATERFIELD MD
AND PONDER BAJ. (1995c). A novel point mutation in the
tyrosine kinase domain of the RET proto-oncogene in sporadic
medullary thyroid carcinoma and in a family with FMTC.
Oncogene, 10, 509-513.

FUKASAWA K, ZHOU R, MATTEN WT, ARMSTRONG AJ, DAAR I,

OSKARSSON M, SATHYANARAYANA BK, MACLVOR L, WOOD
TG AND VANDE WOUDE GF. (1995). Mutagenic analysis of
functional domains of the mos proto-oncogene and identification
of the sites important for MAPK activation and DNA binding.
Oncogene, 11, 1447 - 1457.

HOFSTRA RMW, LANDSVATER RM, CECCHERINI I, STULP RP,

STEELWAGEN T, LUO Y, PASINI B, HOPPENER JWM, PLOOS VAN
AMSTEL HK, ROMEO G, LIPS CJM AND BUYS CHCM. (1994). A
mutation in the RET proto-oncogene associated with multiple
endocrine neoplasia type 2B and sporadic medullary thyroid
carcinoma. Nature, 367, 375-376.

References

ATTIE T, PELET A, EDERY P, ENG C, MULLIGAN LM, AMIEL J,

BOUTRAND L, BELDJORD C, NIHOUL-FEKETE C, MUNNICH A,
PONDER BAJ AND LYONNET S. (1995). Diversity of RET proto-
oncogene mutations in familial and sporadic Hirschsprung
disease. Hum. Mol. Genet., 4, 1381 - 1386.

BELDJORD B, DESCLAUX-ARRAMOND F, RAFFIN-SANSON M,

CORVOL J-C, DE KEYSER Y, LUTON J-P, PLOUIN P-F AND
BERTAGNA X. (1995). The RET proto-oncogene in sporadic
pheochromocytomas: frequent MEN 2-like mutations and new
molecular defects. J. Clin. Endocrinol. Metab., 80, 2063-2068.

BOLINO A, SCHUFFENECKER I, LUO Y, SERI M, SILENGO M,

TOCCO T, CHABRIER G, HOUDENT C, MURAT A, SCHLUMBER-
GER M, TOURNIAIRE J, LENOIR GM AND ROMEO G. (1995).
RET mutations in exons 13 and 14 of FMTC patients. Oncogene,
10, 2415-2419.

CARLSON KM, DOU S, CHI D, SCAVARDA N, TOSHIMA K,

JACKSON CE, WELLS SA, GOODFELLOW PJ AND DONIS-
KELLER H. (1994). Single missense mutation in the tyrosine
kinase catalytic domain of the RET protooncogene is associated
with multiple endocrine neoplasia type 2B. Proc. Natl Acad. Sci.
USA, 91, 1579-1583.

COLLEDGE WH, CARLTON MBL, UDY GB AND EVANS MJ. (1994).

Disruption of c-mos causes parthenogenetic development of
unfertilized mouse eggs. Nature, 370, 65- 68.

DAVENPORT AP. (1996). Distribution of endothelin receptors. In

Endothelins in Biology and Medicine, Miller R, Pelton JT and
Huggins J. (eds) CRC Press: Florida (in press).

DAVENPORT AP, MAGUIRE JJ AND KARET FE. (1994). Endothelin

receptors and their subtypes. In Endothelin: Role in Health and
Disease, Gulati A. (ed.) pp. 17 - 30. Harwood Academic Publish-
ers: Amsterdam.

DONIS-KELLER H, DOU S, CHI D, CARLSON KM, TOSHIMA K,

LAIRMORE TC, HOWE JR, MOLEY JF, GOODFELLOW P AND
WELLS SA. (1993). Mutations in the RET proto-oncogene are
associated with MEN 2A and FMTC. Hum. Mol. Genet., 2, 851 -
856.

ENG C, SMITH DP, MULLIGAN LM, NAGAI MA, HEALEY CS,

PONDER MA, GARDNER E, SCHEUMANN GFW, JACKSON CE,
TUNNACLIFFE A AND PONDER BAJ. (1994). Point mutation
within the tyrosine kinase domain of the RET proto-oncogene in
multiple endocrine neoplasia type 2B and related sporadic
tumours. Hum. Mol. Genet., 3, 237-241.

ENG C, CROSSEY PA, MULLIGAN LM, HEALEY CS, HOUGHTON C,

PROWSE A, CHEW SL, DAHIA PLM, O'RIORDAN JLH, TOLEDO
SPA, SMITH DP, MAHER ER AND PONDER BAJ. (1995a).
Mutations of the RET proto-oncogene and the von Hippel -
Lindau disease tumour suppressor gene in sporadic and
syndromic phaeochromocytoma. J. Med. Genet., 32, 934-937.

ENG C, MULLIGAN LM, SMITH DP, HEALEY CS, FRILLING A,

RAUE F, NEUMANN HPH, PFRAGNER R, BEHMEL A, LORENZO
MJ, STONEHOUSE TJ, PONDER MA AND PONDER BAJ. (1995b).
Mutation of the RET proto-oncogene in sporadic medullary
thyroid carcinoma. Genes Chrom. Cancer, 12, 209 - 212.

ENG C, SMITH DP, MULLIGAN LM, HEALEY CS, ZVELEBIL MJ,

STONEHOUSE TJ, PONDER MA, JACKSON CE, WATERFIELD MD
AND PONDER BAJ. (1995c). A novel point mutation in the
tyrosine kinase domain of the RET proto-oncogene in sporadic
medullary thyroid carcinoma and in a family with FMTC.
Oncogene, 10, 509-513.

FUKASAWA K, ZHOU R, MATTEN WT, ARMSTRONG AJ, DAAR I,

OSKARSSON M, SATHYANARAYANA BK, MACLVOR L, WOOD
TG AND VANDE WOUDE GF. (1995). Mutagenic analysis of
functional domains of the mos proto-oncogene and identification
of the sites important for MAPK activation and DNA binding.
Oncogene, 11, 1447- 1457.

HOFSTRA RMW, LANDSVATER RM, CECCHERINI I, STULP RP,

STEELWAGEN T, LUO Y, PASINI B, HOPPENER JWM, PLOOS VAN
AMSTEL HK, ROMEO G, LIPS CJM AND BUYS CHCM. (1994). A
mutation in the RET proto-oncogene associated with multiple
endocrine neoplasia type 2B and sporadic medullary thyroid
carcinoma. Nature, 367, 375-376.

c-mos and ENRB mutations in MTC and phaeochromocytoma

C Eng et at                                                       03

IA I1

KOMMINOTH P, KUNZ EK, MATIAS-GUIU X, HIORT 0, CHRIS-

TENSEN G, COLOMER A, ROTH J AND HEITZ PU. (1995).
Analysis of RET proto-oncogene point mutations distinguishes
heritable from nonheritable medullary thyroid carcinomas.
Cancer, 76, 479-489.

LINDOR NM, HONCHEL R, KHOSLA S AND THIBODEAU SN. (1995).

Mutations in the RET protooncogene in sporadic pheochromo-
cytomas. J. Clin. Endocrinol. Metab., 80, 627-629.

MATHEW CGP, SMITH BA, THORP K, WONG Z, ROYLE NJ,

JEFFREYS AJ AND PONDER BAJ. (1987). Deletion of genes on
chromosome 1 in endocrine neoplasia. Nature, 328, 524- 526.

MULLIGAN LM, KWOK JBJ, HEALEY CS, ELSDON MJ, ENG C,

GARDNER E, LOVE DR, MOLE SE, MOORE JK, PAPI L, PONDER
MA, TELENIUS H, TUNNACLIFFE A AND PONDER BAJ. (1993).
Germline mutations of the RET proto-oncogene in multiple
endocrine neoplasia type 2A. Nature, 363, 458 -460.

MULLIGAN LM, ENG C, HEALEY CS, PONDER MA, FELDMAN GL,

LI P, JACKSON CE AND PONDER BAJ. (1994). A de novo mutation
of the RETproto-oncogene in a patient with MEN 2A. Hum. Mol.
Genet., 3, 1007-1008.

MULLIGAN LM, MARSH DJ, ROBINSON BG, SCHUFFENECKER I,

ZEDENIUS J, LIPS CJM, GAGEL RF, TAKAI S-I, NOLL WW, FINK
M, RAUE F, LACROIX A, THIBODEAU SN, FRILLING A, PONDER
BAJ AND ENG C. (1995). Genotype-phenotype correlation in
MEN 2: report of the International RET Mutation Consortium.
J. Intern. Med., 238, 343-346.

OKAMOTO E AND UEDA T. (1967). Embryogenesis of intramural

ganglia of the gut and its relation to Hirschsprung disease. J.
Pediatr. Surg., 10, 437-443.

PUFFENBERGER EG, HOSODA K, WASHINGTON SS, NAKAO K, DE

WIT D, YANAGISAWA M AND CHAKRAVARTI A. (1995). A
missense mutation of the endothelin B receptor gene in multigenic
Hirschsprung's disease. Cell, 79, 1257- 1266.

SAGATA N, OSKARSSON M, COPELAND T, BRUMBAUGH J AND

VANDE WOUDE GF. (1988). Function of c-mos proto-oncogene
product in meiotic maturation in Xenopus oocytes. Nature, 335,
519- 525.

SCHULZ N, PROPST F, ROSENBERG MP, LINNOILA RI, PAULES RS,

KOVATCH R, OGISO Y AND VANDE WOUDE GF. (1992).
Pheochromocytomas and c-cell thyroid neoplasms in transgenic
c-mos mice: a model for the human multiple endocrine neoplasia
type 2 syndrome. Cancer Res., 52, 450-455.

YEW N, STROBEL M AND VANDE WOUDE GF. (1993). Mos and the

cell cycle: the molecular basis of the transformed phenotype. Curr.
Opin. Genet. Devel., 3, 19-25.

ZEDENIUS J, WALLIN G, HAMBERGER B, NORDENSKJOLD M,

WEBER G AND LARSSON C. (1994). Somatic and MEN 2A de
novo mutations identified in the RET proto-oncogene by
screening of sporadic MTCs. Hum. Mol. Genet., 3, 1259-1262.

				


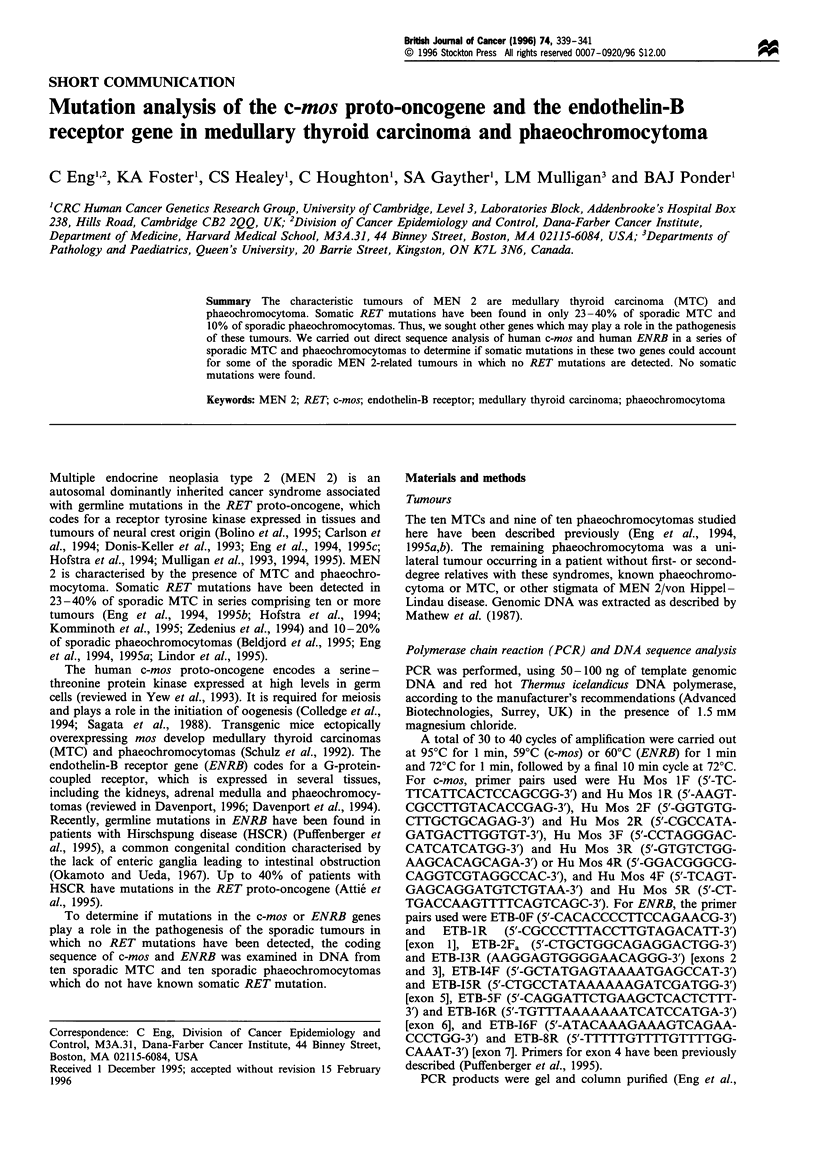

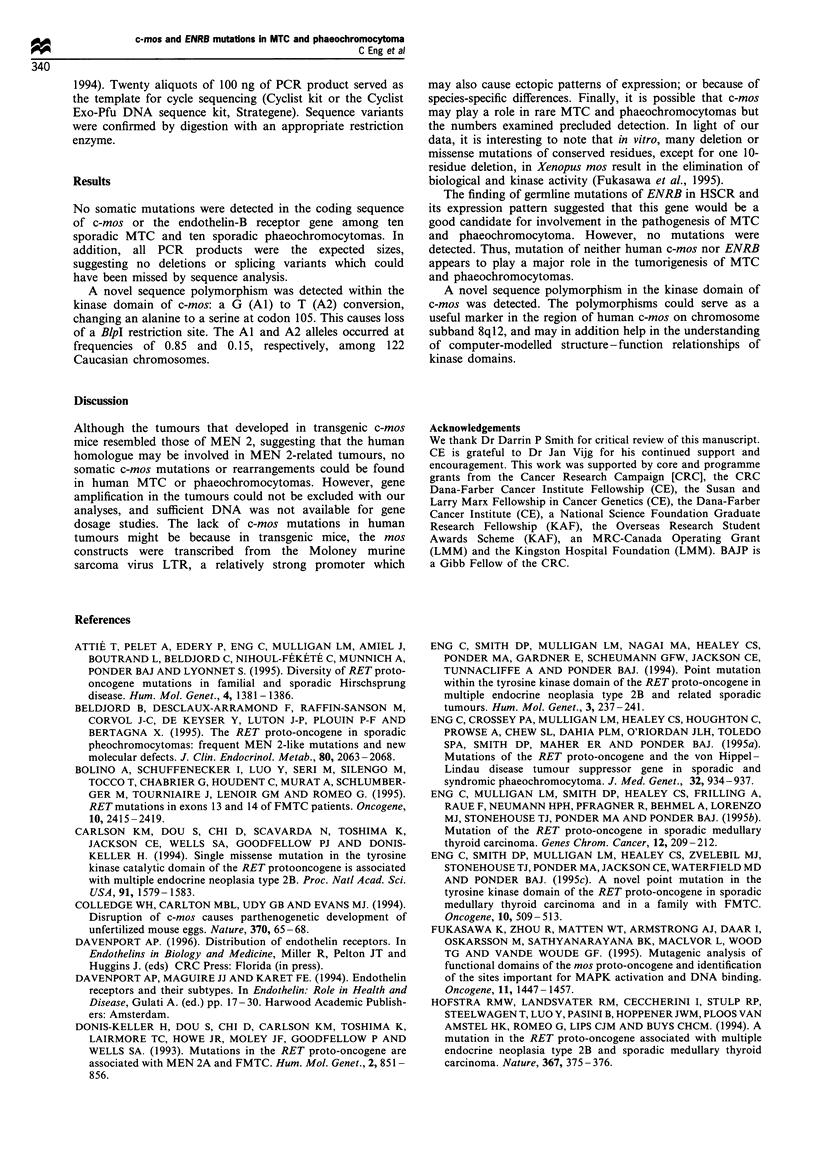

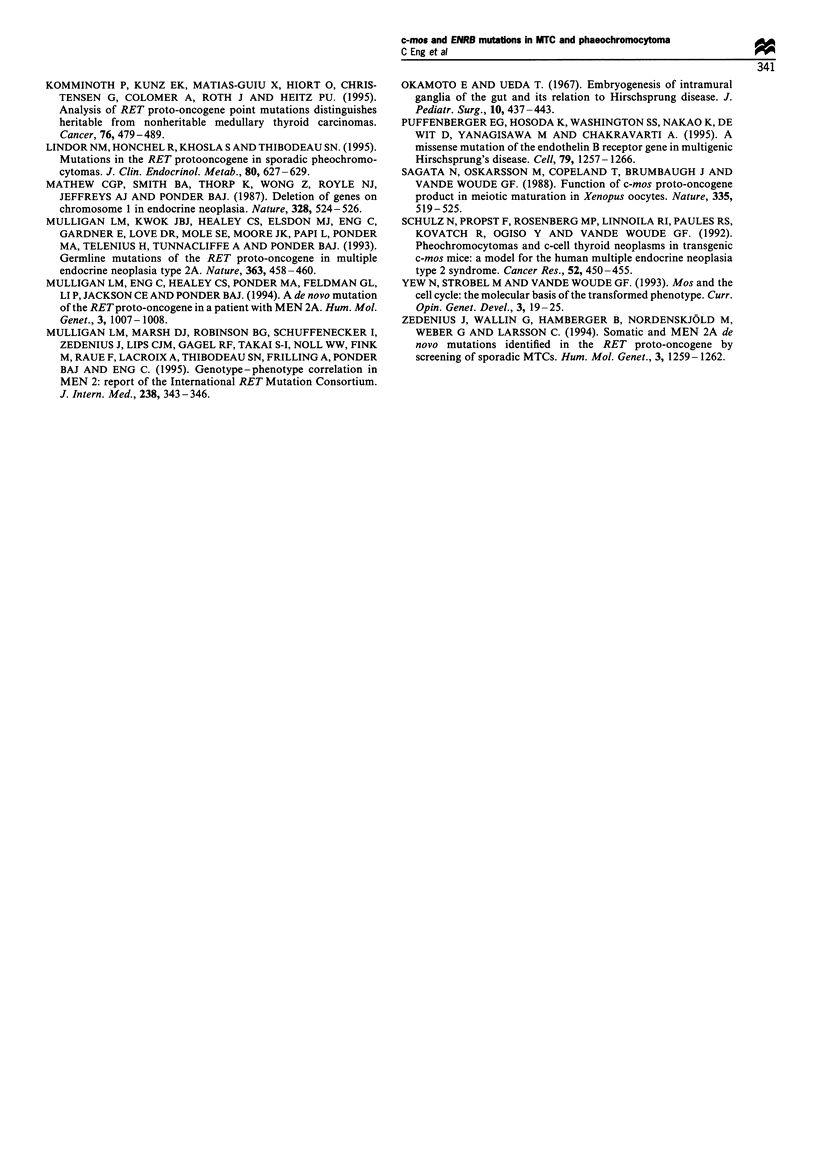

